# Supplementary material for: A Small Molecule-Controlled Cas9 Repressible System
Source: Mol Ther Nucleic Acids. 2020 Jan 10;19:922–32. doi: 10.1016/j.omtn.2019.12.026 (PMC7063486; doi:10.1016/j.omtn.2019.12.026)
Supplement: Document S1. Figures S1–S5 and Tables S1–S4 [file mmc1.pdf]

OMTN, Volume *19*

Supplemental Information

A Small Molecule-Controlled Cas9 Repressible System

Youjun Wu, Lu Yang, Tammy Chang, Fouad Kandeel, and Jiing-Kuan Yee

Supplemental information

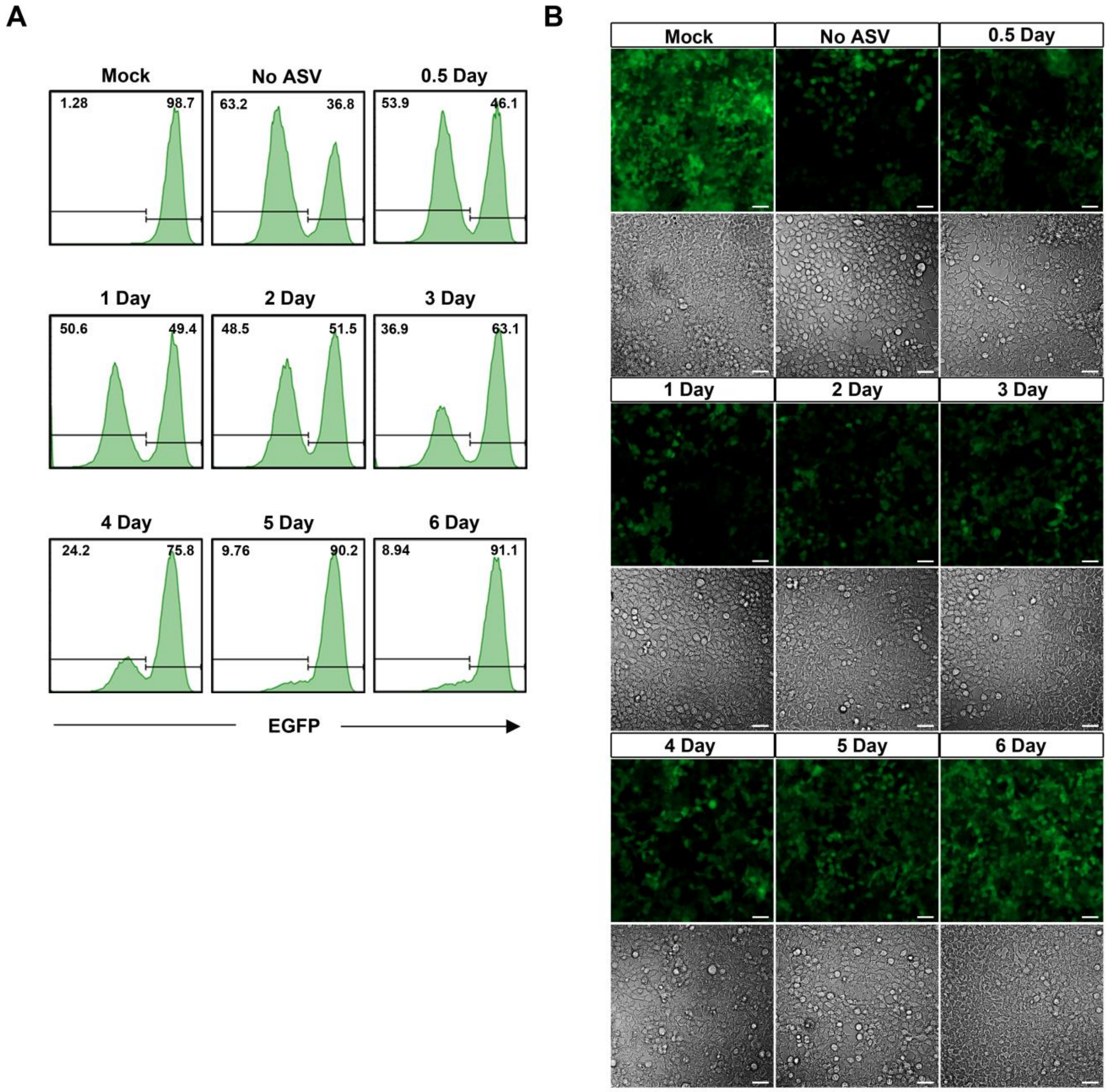

Figure S1. Restoration of the *EGFP* gene knockout activity following ASV removal. HEK293T cells co-transfected with plasmids expressing NC-SMASh Cas9 and a sgRNA against the *EGFP* gene in the presence of 20 μM ASV were washed at indicated time points. The *EGFP* gene knockout efficiency was measured by FACS in (A) and fluorescence microscopy in (B) 6 days post-transfection. Data shown is a representative of three independent experiments. Scale bar = 50 μm.

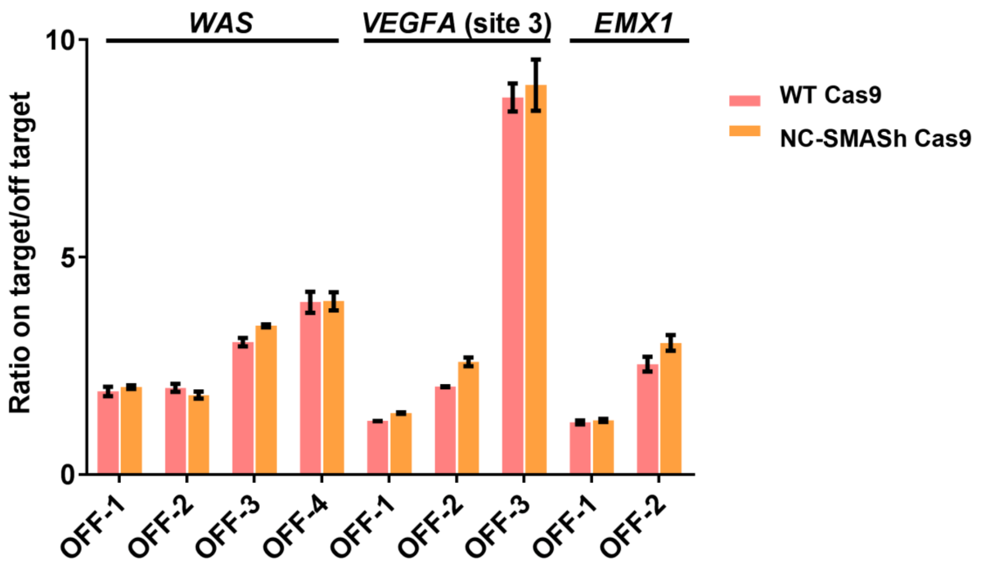

Figure S2. Similar targeting specificity between WT Cas9 and NC-SMASh Cas9 in the absence of ASV. HEK293T cells were co-transfected with plasmids expressing either WT Cas9 or NC-SMASh Cas9 and the sgRNA targeting the *WAS*, *VEGFA* or *EMX1* locus as indicated. On- and off-target sites were PCR amplified and subjected to deep sequencing. The on/off target site ratio was calculated with the indel frequency of each locus. Data represents mean ± SEM from two independent deep-sequencing experiments.

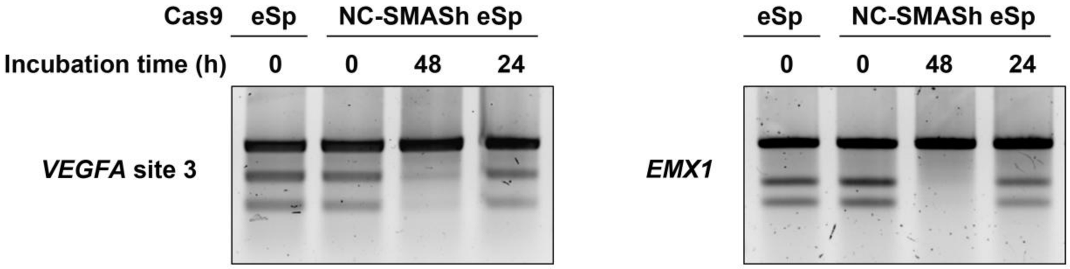

Figure S3. Reversible gene editing with NC-SMASh eSpCas9 in response to ASV treatment. HEK293T cells were co-transfected with plasmids expressing either eSpCas9 or NC-SMASh eSpCas9 and the sgRNA targeting the *VEGFA* site 3 or *EMX1* locus as indicated. The transfected cultures were treated with 20 µM ASV for 48 h or treated for the first 24 h followed by ASV removal and incubation for an additional 24 h in fresh medium without ASV. All cultures were harvested 48 h after transfection and the isolated genomic DNA was subjected to the Surveyor assay to measure indel formation.

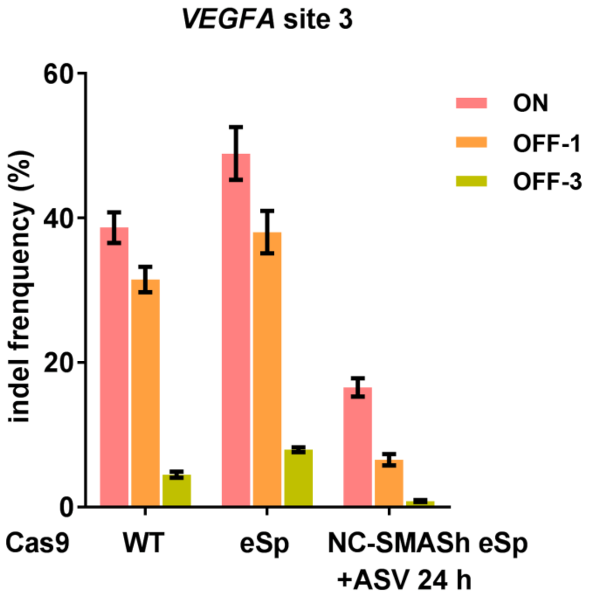

Figure S4. Increased targeting specificity of NC-SMASh Cas9. HEK293T cells were co-transfected with plasmids expressing WT Cas9, eSpCas9, or NC-SMASh eSpCas9 and the sgRNA targeting the *VEGFA* site 3 locus. Cells expressing NC-SMASh eSpCas9 were treated with 20 µM ASV immediately after transfection for 24 h followed by its removal and incubation in fresh medium for an additional 24 h. On- and off-target sites were PCR amplified and subjected to deep sequencing. Data represent mean ± SEM from two independent deep-sequencing experiments.

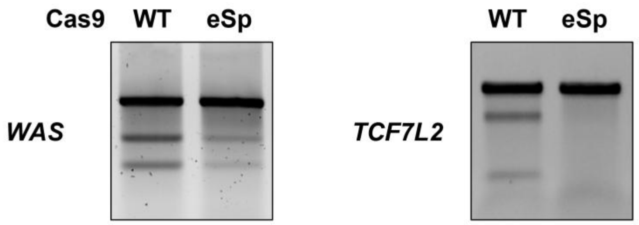

Figure S5. Genomic loci resistant to the cleavage by eSpCas9. HEK293T cells were co-transfected with plasmids expressing WT Cas9 or eSPCas9 and the sgRNA targeting either the *WAS* or *TCF7L2* locus. Surveyor assay was performed to detect indel formation.

Table S1. sgRNA sequences used in this paper.

Gene	Sequence	PAM
WAS	TGGATGGAGGAATGAGGAGT	TGG
PCSK1	ATAAGTGTGTGGTCCCAAGT	TGG
EGFP	GGCGAGGGCGATGCCACCTA	CGG
VEGFA	GGTGAGTGAGTGTGTGCGTG	TGG
EMX1	GAGTCCGAGCAGAAGAAGAA	GGG
TCF7L2	TATAATTTAATTGCCGTGTG	AGG

Table S2. Primers for plasmid construction

Plasmid	Primer set	Product size
C-SMASh Cas9	FP1: CAGCTAGCTAGCGATGAGATGGAAGAGTGCTC<br>RP1: ACAGCGACCGGTTCAGTAGAGAACCTCCCTGTC	939
	FP2: TCGAGCAAATAAGCGAATTCTCC<br>RP2: ACAGCGGCTAGCCACCTTCCTCTTCTTCTTGGGGTC	341
NC-SMASh Cas9	FP3: CAGAAGGCTCGAGGACAAGAAGTACTCCATTGG<br>RP3: CATTACTAAAGATCTCCTGCAGGTAGC	275
	FP4: TGCTCTAGAATGGATTACAAGGATGACGACG<br>RP4: CAGAAGGCTCGAGGCCCATTGAGCATGGCACG	931

Table S3. Primers used for Surveyor assay

Target Gene	Primer set	Expected PCR product (bp)	Expected cleaved products (bp)
WAS	FP: AAAGGAAGTTGGGCAGAGGTGAGT<br>RP:<br>CCATCGATTGTGTGTTGGATGGTCATGGAGGT	838	499; 339
PCSK1	FP: GCCCACACAAGAGAACCCTAA<br>RP: TTGAGCTCATCCCCTTCACAT	540	391; 149
VEGFA	FP: CTCACTTTGATGTCTGCAGGCCA<br>RP: CGAGGAGGGAGCAGGAAAGTGA	528	333; 195
EMX1	FP: CCATCCCCTTCTGTGAATGT<br>RP: GGAGATTGGAGACACGGAGA	639	272; 367
TCF7L2	FP: TCTTGCTTAGTCACTTTCTGTTTGAACA<br>RP: CAGTGAAGTGCCCAAGCTTCTCA	500	360; 140

Table S4. Primers used for deep-sequencing

Name	Gene	Target sequence	Primer set
WAS ON	*WAS*	TGGATGGAGGAATGAGGAGTTGG	FP:TGCGTGCTGATTCTTCCCTG<br>RP:TGCTCGTCCATCCACATACC
WAS OFF1	*MATN1-AS1*	TGGATGGAGGGATGAGGAGTGGG	FP:TGGCACATAGAGGAGCCTGA<br>RP:GGGGGCTGTTTGTTATGCAC
WAS OFF2	*OSBPL1A*	AGGAGGGAGGAATGGGGAGTTGG	FP:AGTAGGGTTAGGCCACGACA<br>RP:TTCCTATGCAACCATCACCA
WAS OFF3	*MYCN*	GAGGATGGGGAATGAGGAGTAGG	FP:TTGGGCAGGTGGGAGTCAAT<br>RP:CCACTCCTGTTGACCTGCTC
WAS OFF4	*CD70*	CGGACGGAGGAATGGGGAGTGGG	FP:GGCACTGGTCACCTGTTTGA<br>RP:TGAGAGGAGTGCTTGATGAAAAGA
VEGFA ON	*VEGFA* (site 3)	GGTGAGTGAGTGTGTGCGTGTGG	FP:GCGAGCAGCGTCTTCGAGAGTGAGGA<br>RP:GGAGAGGGACACACAGATCTATTGGAA
VEGFA OFF1	*MAX*	AGTGAGTGAGTGTGTGTGTGGGG	FP: AGGGGAGGGGGAAGTCACCGA<br>RP: GGAGGTGGTTCTTGCCCGTG
VEGFA OFF2	*CYTH4*	GCTGAGTGAGTGTATGCGTGTGG	FP: GCCCATTTCTCCTTTGAGGTTCATCC<br>RP: TGGTGGGGACAGCATGTGCAAG
VEGFA OFF3	*TMEM121 (abParts)*	GGTGAGTGAGTGTGTGTGTGAGG	FP: AGGGACCCCTCTGACAGACT<br>RP: AGCCCTCAGACTTCACATTC
EMX1 ON	*EMX1*	GAGTCCGAGCAGAAGAAGAAGGG	FP: CAAAGTACAAACGGCAGAAGC<br>RP: GTTGCCCACCCTAGTCATTG
EMX1 OFF1	*HCN1*	GAGTTAGAGCAGAAGAAGAAAGG	FP: TATCACCTATTTTTTCTGAGGGCTGCT<br>RP: GGGTTACAGAAAGAATAGGGGCTTATG
EMX1 OFF2	*MFAP1*	GAGTCTAAGCAGAAGAAGAAGAG	FP: CACGGCCTTTGCAAATAGAG<br>RP: GGCTTTCACAAGGATGCAGT

Mismathes are colored in red.
