# Supplementary material for: A Small Molecule-Controlled Cas9 Repressible System
Source: Mol Ther Nucleic Acids. 2020 Jan 10;19:922–32. doi: 10.1016/j.omtn.2019.12.026 (PMC7063486; doi:10.1016/j.omtn.2019.12.026)
Supplement: Document S2. Article plus Supplemental Information [file mmc2.pdf]

Molecular Therapy
Nucleic Acids
Original Article

A Small Molecule-Controlled Cas9 Repressible System

Youjun Wu,[1] Lu Yang,[2] Tammy Chang,[1] Fouad Kandeel,[1] and Jiing-Kuan Yee[1]

[1]Department of Translational Research and Cellular Therapeutics, Diabetes and Metabolism Research Institute, Beckman Research Institute of City of Hope, Duarte, CA 91010, USA; [2]Department of Systems Biology, Beckman Research Institute of City of Hope, Monrovia, CA 91016, USA

CRISPR-Cas9 has been developed into a powerful molecular tool for genome engineering, and it has revolutionized the field of biomedical research. Despite the tremendous potential of CRISPR-Cas9 in biomedical research, precise control of CRISPR-Cas9 over the dose and exposure time is important to expand its applications. In this study, we fused Cas9 with a peptide termed small molecule-assisted shut-off (SMASh) consisting of a protease domain and a degron domain derived from hepatitis C virus (HCV). The presence of SMASh allows tight control of the Cas9 stability via a clinically approved HCV protease inhibitor asunaprevir (ASV). We showed that the engineered Cas9 responded to ASV administration and rapidly degraded in a dose- and time-dependent manner. Cas9 degradation was reversible upon ASV removal that restored the gene editing activity. We also showed that limiting the level of Cas9 in cells increased the specificity of gene editing. The SMASh tag therefore provides an effective tool to control Cas9 stability, allowing an improvement in the accuracy, safety, and versatility of the CRISPR-Cas9 system for genome editing and gene regulation studies.

INTRODUCTION

The CRISPR-Cas9 system was discovered in bacteria and archaea, where it works as a self-defense system to protect against invading viruses and foreign nucleic acids.[1–3] The system has now been developed into a powerful molecular tool for genome engineering, and it has revolutionized the field of biomedical research.[4,5] The most well-known type II CRISPR-Cas9 system consists of Cas9 from *Streptococcus pyogenes* (SpCas9) and a single guide RNA (sgRNA) with 20 nt complementary to the genomic target adjacent to a protospacer-adjacent motif (PAM).[6,7] Base paring between the sgRNA and its genomic target directs the Cas9 nuclease to bind and generate double-strand breaks (DSBs) at the intended locus. The DSB is then repaired via non-homologous end joining (NHEJ), leading to the generation of insertions or deletions (indels), or via homology-directed repair (HDR) in the presence of a homologous donor template.[8–10] The properties of CRISPR-Cas9 make it widely applicable to alter the genome from diverse species. These applications facilitate studies to understand gene function and biological processes, and they hold enormous promises for therapeutic treatment of human diseases.[6,7]

Despite the tremendous potential of CRISPR-Cas9, precise control of Cas9 protein over its dose and exposure time is important to expand its applications. CRISPR-Cas9 can generate off-target cleavage at unintended genomic sites and induce gene mutation or genome instability.[11–13] Limiting cell exposure to Cas9 is expected to reduce the off-target effect. For *in vivo* studies, mosaic genome mutations were created in the embryos of mouse and non-human primates due to the persistent activity of Cas9 in dividing cells. Promoting Cas9 degradation in such cases was shown to reduce the mosaicism.[14–16] When nuclease-deficient Cas9 (dCas9) covalently linked with a transcriptional activator or repressor was used to modulate gene expression, tight control of these dCas9-based transcription regulators would facilitate the study of gene function in cells or during development.[17–21] As CRISPR-Cas9 has been proposed to be used *in vivo* for therapeutic treatment of human diseases, precise control of the Cas9 stability would limit its exposure and reduce the risk of eliciting immune responses against the protein.[22–25] With these considerations in mind, a number of strategies have been developed by engineering the Cas9 protein to control its activity or stability. Several approaches use small molecules or optical light to activate functionally dormant Cas9.[26–33] Another approach uses bacteriophage-encoded anti-CRISPR proteins to switch off wild-type (WT) Cas9 activity through inhibition of CRISPR-Cas9 to bind to its genomic target.[34,35] More recently, through the screening of a chemical library, a small molecule that perturbs the binding of CRISPR-Cas9 to DNA has been discovered.[36] In general, these strategies enable conditional modulation of the Cas9 activity, stability, or its interaction with the genomic target. Since no single strategy is sufficiently robust to fulfill the promises of CRISPR-Cas9 in both safety and efficacy, additional approaches to better control the activity and stability of Cas9 are sought.

In the current work, we employed a small molecule-assisted shut-off (SMASh) technique to develop a repressible Cas9 system capable of degrading newly synthesized Cas9 protein rapidly.[37] This technique involves the fusion of the protein of interest with a SMASh tag consisting of a protease domain and a degron derived from hepatitis C virus (HCV). The protease self-cleaves to remove the SMASh tag from the fusion protein in the absence of HCV protease inhibitors.

Received 10 December 2019; accepted 21 December 2019;
https://doi.org/10.1016/j.omtn.2019.12.026.

Correspondence: Jiing-Kuan Yee, Department of Translational Research and Cellular Therapeutics, Diabetes and Metabolism Research Institute, Beckman Research Institute of City of Hope, Duarte, CA 91010, USA.
E-mail: jyee@coh.org

 © 2020 The Author(s).
This is an open access article under the CC BY license (http://creativecommons.org/licenses/by/4.0/).

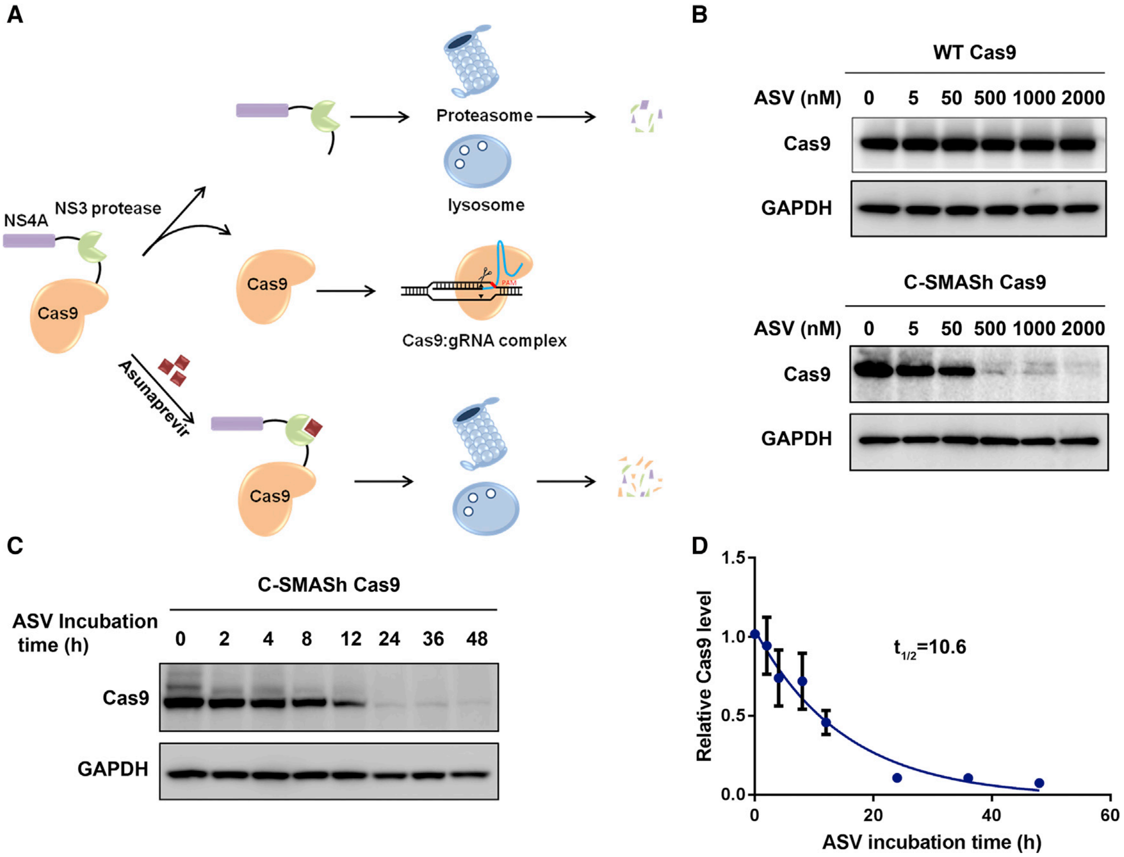

Figure 1. Regulation of the Cas9 Stability by the SMASh Tag

(A) Schematic of controlling the Cas9 stability via the SMASh tag. In the absence of ASV, the NS3 protease self-cleaves and removes the SMASh tag from the fusion protein and thus preserves the Cas9 stability. ASV administration inhibits the NS3 protease activity, leading to degradation of the fusion protein by proteasome and lysosome. (B) Western blot analysis of HEK293T cells expressing either WT Cas9 or C-SMASh Cas9 in the absence or presence of ASV at the indicated concentration for 24 h. GAPDH serves as the loading control. (C) Western blot analysis to measure the degradation of Cas9 generated before ASV administration. HEK293T cells were transfected with the expression plasmid for C-SMASh Cas9 in the absence of ASV for 24 h. Cells were treated with 1 μM ASV to block the accumulation of newly synthesized Cas9. The level of Cas9 was then monitored at various time points after the ASV treatment. GAPDH serves as the loading control. (D) Calculation of Cas9 half-life with the data from (C). Band intensity of Cas9 was quantified, normalized to GAPDH, and divided by the signal at 0 h. Western blot study was performed three times, and error bars represent SEM. Half-life of Cas9 was calculated by fitting a curve using nonlinear regression and a one-phase exponential decay equation.

The original stability of the protein is therefore preserved. Adding a protease inhibitor prevents the removal of the SMASh tag, leading to rapid degradation of the fusion protein due to the presence of the degron in the SMASh tag.[37] We engineered Cas9 to fuse with the SMASh tag and showed regulated Cas9 stability by the protease inhibitor in a dose- and time-dependent manner. We also demonstrated that by modulating the level of Cas9 in cells, this system increased the specificity of gene editing. The system we present therefore confers multidimensional control of Cas9 at the post-translational level and improves its versatility as a tool to perform genome editing and to study gene regulation.

RESULTS

Modulation of Cas9 Stability Using SMASh

A SMASh tag comprises the HCV nonstructural protein 3 (NS3) protease domain followed by the HCV nonstructural protein 4a (NS4A) that acts as a degron.[37] In the absence of the protease inhibitor, a SMASh tag removes itself from the fusion protein by default. However, the presence of an HCV protease inhibitor such as asunaprevir (ASV) blocks the protease activity, leading to the degradation of the fusion protein through the proteasome and autophagolysosome pathways.[37] To evaluate the effect of a SMASh tag on Cas9, we fused a SMASh tag in *cis* to the C terminus of Cas9 (C-SMASh Cas9) (Figure 1A) and measured the level of the Cas9 protein in response to ASV treatment. We transfected HEK293T cells with the expression plasmid for C-SMASh Cas9 in the presence of ASV at various concentrations. In the absence of ASV, tagging Cas9 with the HCV degron did not affect its stability (Figure 1B). In response to increasing concentrations of ASV, the level of Cas9 displayed a dose-dependent reduction, with a dramatic decrease at ASV concentrations >50 nM (Figure 1B, lower panel). In contrast, the level of WT Cas9 was not affected by ASV treatment (Figure 1B, upper panel), indicating that

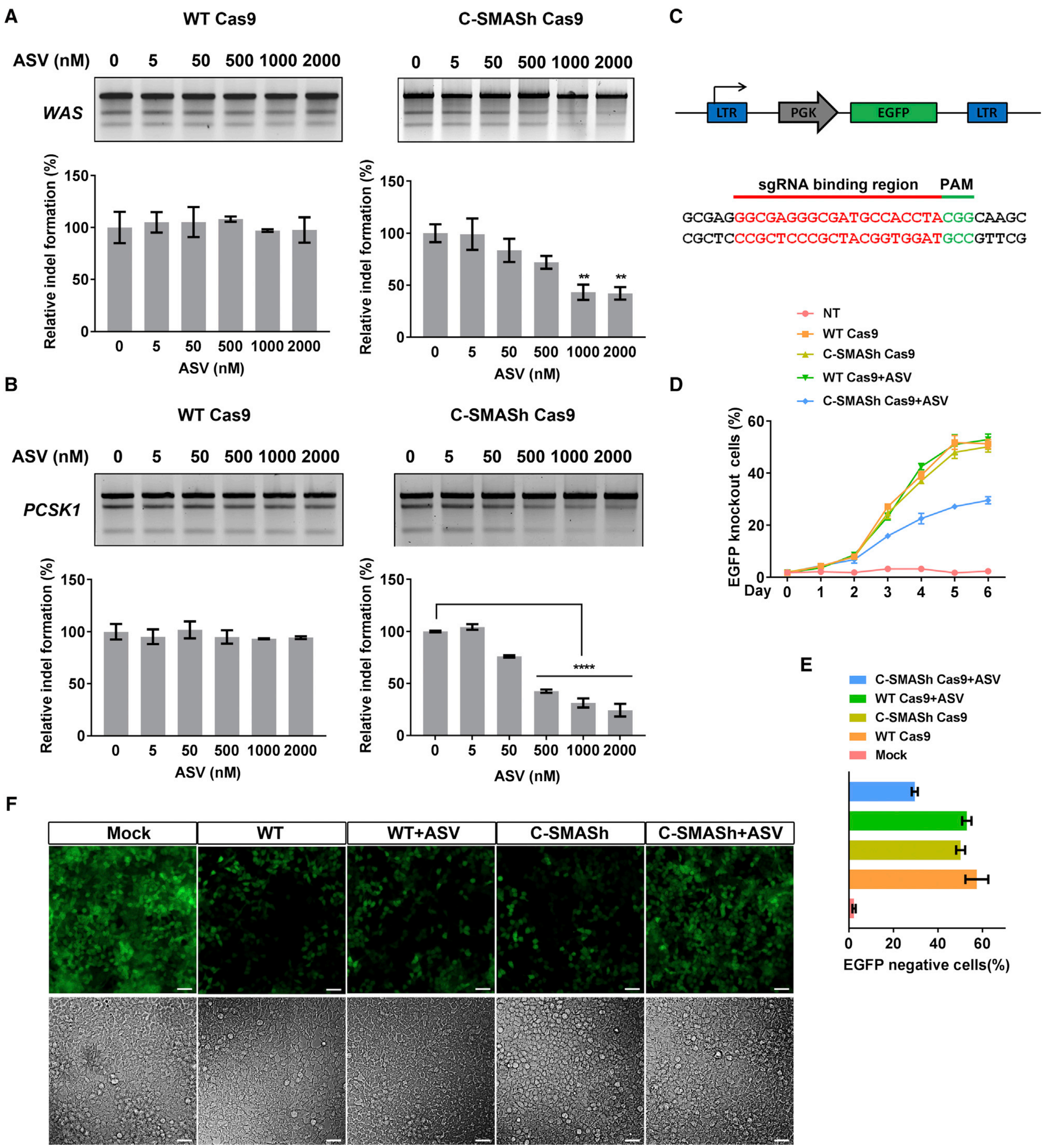

Figure 2. SMASh-Mediated Control of Genome Editing

(A and B) Dose-dependent control of genome editing by C-SMASh Cas9. HEK293T cells were co-transfected with the expression plasmid for either WT Cas9 or C-SMASh Cas9 and the expression plasmid for the indicated sgRNA without or with ASV at the indicated concentration for 48 h. Indel-forming efficiencies at the *WAS* locus in (A) and the *PCSK1* locus in (B) were evaluated with the Surveyor assay. The percentage of indels of each sample was quantified as described in Materials and Methods, normalized to that of the control without ASV, and expressed as relative indel formation. Bars represent the mean ± SEM from three independent experiments. **$p < 0.01$, ****$p < 0.0001$ by one-way ANOVA and Dunnett's test for multiple comparison versus non-drug control. (C) Structure of the *EGFP* lentiviral vector and the sgRNA target sequence in the *EGFP* gene. (D) *EGFP* knockout activity of WT Cas9 and C-SMASh Cas9 in the presence of 2000 nM ASV. Knockout efficiency was analyzed using flow cytometry from day 1 to day

(legend continued on next page)

ASV selectively degraded C-SMASh Cas9. Because ASV only blocks the accumulation of newly synthesized protein without affecting protein already produced before protease inhibitor administration, this system allows measurement of protein half-life via its decay rate in the cell. We quantified the protein level of Cas9 in HEK293T cells transiently expressing C-SMASh Cas9 for 24 h, followed by ASV treatment for various times (2–48 h). Western blot demonstrated a progressive decrease in Cas9 protein upon ASV incubation (Figure 1C), and the half-life was estimated to be ~10.6 h based on a time-course protein analysis (Figure 1D). The half-life of Cas9 determined with this approach is shorter by several hours than the half-life determined by a previous study that measured Cas9 half-life by transfecting the CRISPR-Cas9 ribonucleoprotein (RNP) complex into human myelogenous leukemia K562 cells.[38] The discrepancy could be due to the formation of a stable RNP complex before cell delivery that protects the Cas9 protein from degradation by cellular proteases. Use of different cell lines for measurement could also be attributed to the observed difference in the Cas9 half-life.

Regulation of the Genome Editing Activity of C-SMASh Cas9

To study genome editing by C-SMASh Cas9, we used the Surveyor assay to measure indel formation at two different loci, the Wiskott-Aldrich syndrome (*WAS*) gene and the proprotein convertase subtilisin/kexin type 1 (*PCSK1*) gene. In the absence of ASV, WT Cas9 and C-SMASh Cas9 showed a similar indel-forming efficiency (Figures 2A and 2B). Exposure to ASV did not affect indels generated by WT Cas9, whereas increasing concentrations of ASV led to a progressive decrease in indels generated by C-SMASh Cas9 (Figures 2A and 2B). Notably, higher doses of ASV (ranging from 500 to 2,000 nM) yielded greater reduction in indel formation, consistent with the result in Figure 1B that ASV at these concentrations led to a dramatic decrease in intracellular Cas9 protein. To further confirm the regulation of the Cas9 activity by the SMASh tag, we performed an enhanced green fluorescence protein (*EGFP*) gene disruption assay. A HEK293T clone bearing a single copy of the integrated *EGFP* gene was co-transfected with expression plasmids encoding WT or C-SMASh Cas9 and a sgRNA targeting the coding region of *EGFP* (Figure 2C). In the absence of ASV, both WT and C-SMASh Cas9 gave rise to increasing percentages of *EGFP* knockout cells as analyzed by fluorescence-activated cell sorting (FACS), which plateaued at 6 days post-transfection (Figure 2D). Quantification of the EGFP-negative cell fraction on day 6 showed that ASV at 2,000 nM had no effect on the efficiency of *EGFP* knockout generated by WT Cas9 (Figure 2E). In contrast, the ability of C-SMASh Cas9 to knockout the *EGFP* gene was significantly compromised in response to ASV treatment (Figure 2E). A similar result was observed with fluorescence microscopy; the fraction of EGFP knockout cells was increased with WT Cas9 relative to the mock control regardless of whether ASV was present, whereas the fraction was significantly reduced with C-SMASh Cas9 in the presence of ASV (Figure 2F).Collectively, our data show that use of a SMASh tag to control Cas9 protein stability allows drug-mediated regulation of genome editing.

Optimization of SMASh-Tagged Cas9

Although the continuous presence of 2,000 nM ASV led to degradation of a majority of the intracellular C-SMASh Cas9, traces of indel remained detectable in the ASV-treated cells (Figures 2A and 2B). We thus sought to improve this system by fusing the SMASh tag to both the N and C termini of Cas9 (NC-SMASh Cas9). In the presence of ASV, Cas9 protein was efficiently degraded in the HEK293T cells transfected with the NC-SMASh Cas9 expression plasmid 24 h post-transfection whereas residual Cas9 protein derived from C-SMASh Cas9 remained detectable under the same condition (Figure 3A). We also observed that the level of NC-SMASh Cas9 in the absence of ASV was reduced compared with that of C-SMASh Cas9 or WT Cas9 (Figure 3A). This may be because self-removal of the two SMASh tags is not as efficient as that of the single C-SMASh tag. However, despite the reduction in the basal Cas9 level, the efficiency to edit the *PCSK1* and *WAS* loci by NC-SMASh Cas9 was similar to C-SMASh Cas9 in the absence of ASV (Figure 3B). This likely reflects the fact that the current method for Cas9 expression plasmid transfection led to excessive production of the Cas9 nuclease. However, upon ASV addition, reduction in the editing efficiency was more pronounced with NC-SMASh Cas9 than C-SMASh Cas9 at both the *WAS* and *PCSK1* loci (Figure 3B). Consistent with this observation, the efficiency of disrupting the *EGFP* gene with NC-SMASh Cas9 was more effectively blocked by ASV than that with C-SMASh Cas9 (Figure 3C). Based on these results, NC-SMASh Cas9 was used for our subsequent studies since it demonstrated more sensitive responses to ASV treatment.

Reversible Regulation of Genome Editing by NC-SMASh Cas9

To determine whether the SMASh tag allows limited Cas9 exposure, we measured the Cas9 gene editing activity after ASV removal. ASV at a concentration of 2 μM was applied to suppress genome editing right after transfection of the expression plasmids for the NC-SMASh Cas9 and a sgRNA targeting the *WAS* gene. ASV was removed 24 h later and the efficiency of indel formation was determined 48 h post-transfection by the Surveyor assay. As shown in Figure 4A, indel formation was significantly reduced in the continuous presence of ASV for 48 h whereas removal of ASV at 24 h after transfection restored ~74% of the indel relative to the control without ASV treatment. Since residual indel remained visible in cells treated with 2 μM ASV for 48 h, we increased the concentration of ASV to 20 μM to completely shut off the Cas9 activity. The increase of ASV effectively blocked genome editing of NC-SMASh Cas9 at the *WAS* locus (Figure 4A, right). However, the gene editing activity was effectively restored to ~69%

6 after transfection of HEK293T-GFP cells with plasmids expressing Cas9 and the sgRNA targeting the *EGFP* gene shown in (C). Error bars represent SEM from three independent experiments. (E) Quantification of the fraction of *EGFP* knockout cells on day 6 after transfection based on the data shown in (D). Bars represent the mean ± SEM from three independent experiments. (F) Fluorescence (top) and bright-field (bottom) images of HEK293T-GFP cells 6 days after transfection with the expressing plasmids for Cas9 as indicated and the *EGFP* sgRNA without or with 2000 nM ASV. Scale bars, 50 μm.

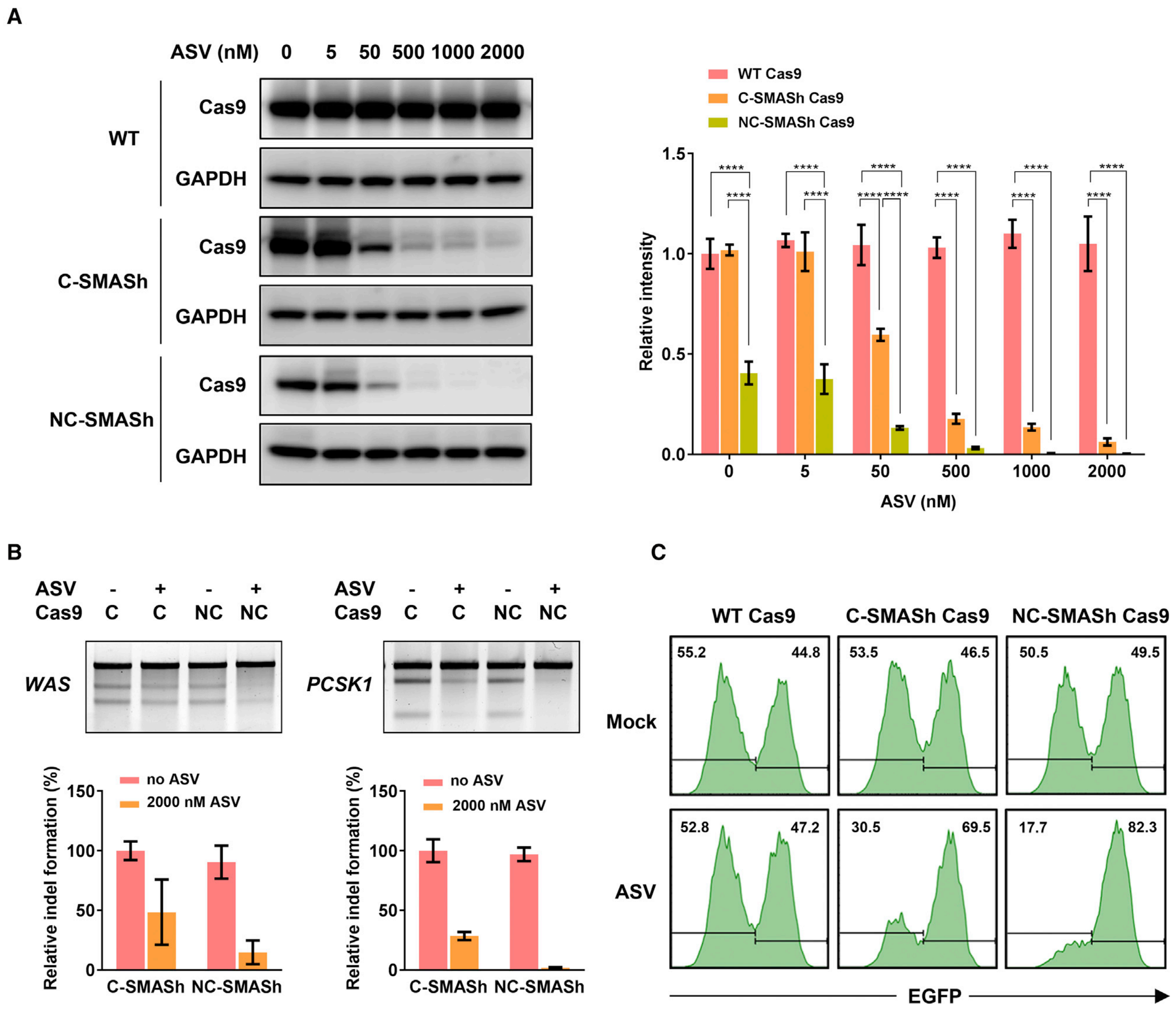

Figure 3. Improved Control of Gene Editing with NC-SMASh Cas9

(A) Western blot analysis of Cas9 in HEK293T cells transfected with the plasmid expressing WT Cas9, C-SMASh Cas9, or NC-SMASh Cas9 with and without ASV treatment for 24 h. Band intensity of Cas9 was quantified and normalized to that of WT Cas9 without ASV and expressed as relative intensity shown on the right. Bars represent the mean ± SEM from three independent experiments. GAPDH serves as the loading control. Data were analyzed by two-way ANOVA with Tukey's *post hoc* test. ****$p < 0.0001$. (B) Inhibition of genome editing with C-SMASh Cas9 and NC-SMASh Cas9 by ASV. Surveyor assay was performed to detect indel formed by C-SMASh Cas9 and NC-SMASh Cas9 with and without 2,000 nM ASV at the *WAS* locus and the *PCSK1* locus. Relative indel forming efficiency was determined as described in Figures 2A and 2B. Data were presented as mean ± SEM from three independent experiments. (C) FACS analysis of the *EGFP* knockout efficiency by the indicated Cas9 protein with and without 2,000 nM ASV. *EGFP* knockout cells were quantified on day 6 after transfection with Cas9- and sgRNA-expressing plasmids.

of the control after ASV removal (Figure 4A, right). Western blot showed a progressive accumulation of the Cas9 protein upon ASV removal, reaching a level of ~38% of the control without any ASV treatment (Figure 4B). The lack of further Cas9 accumulation from 8 to 24 h after ASV removal may be caused by the gradual degradation of the transiently transfected plasmid DNA for NC-SMASh Cas9. Despite this reduction in the Cas9 level, indel formation remained at a level of ~69% relative to the control without ASV, again suggesting that Cas9 was in excess for genome editing. To optimize the timing for reversible modulation of the NC-SMASh Cas9 gene editing activity, *EGFP* knockout efficiency was assessed in HEK293T-EGFP cells by co-transfecting plasmids encoding NC-SMASh Cas9 and the sgRNA targeting the *EGFP* gene. ASV was added from the beginning and removed at different time points, and the fraction of EGFP knockout cells was monitored by fluorescence microscopy and quantified by FACS on day 6 after transfection (Figure 4C; Figure S1). The efficiency of *EGFP* knockout upon ASV removal within 2 days post-transfection was restored to >75% of the control without ASV

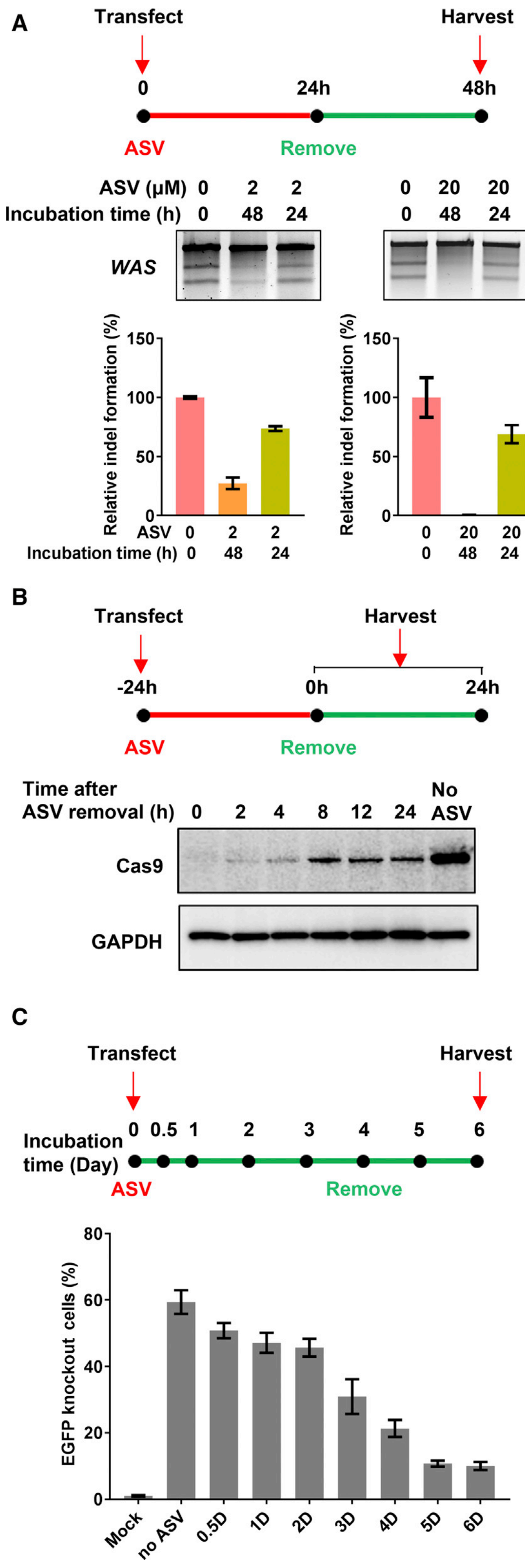

Figure 4. Reversible Control of Genome Editing with NC-SMASh Cas9

(A) Restoration of genome editing with NC-SMASh Cas9 by ASV removal. Plasmids expressing NC-SMASh Cas9 and the sgRNA targeting the *WAS* locus were co-transfected into HEK293T cells in the presence of 2 or 20 μM ASV for 24 h. Cells were washed and incubated in fresh medium without ASV for an additional 24 h. Indel-forming efficiency was determined using the Surveyor assay and normalized to the control without ASV as described in Figures 2A and 2B. Bars represent mean ± SEM from three independent experiments. (B) Restoration of Cas9 by ASV removal. HEK293T cells transfected with the expression plasmid for NC-SMASh Cas9 were cultured for 24 h in the presence of 20 μM ASV, followed by wash and incubation in fresh medium without ASV for an additional 24 h. Cas9 was detected by western blot at the time point indicated after ASV removal. GAPDH serves as the loading control. (C) Reversible *EGFP* gene knockout by Cas9 upon ASV removal. HEK293T cells were co-transfected with the expression plasmids for NC-SMASh Cas9 and the sgRNA against the *EGFP* gene in the presence of 20 μM ASV. Cells were washed at the indicated time point and incubated in fresh medium without ASV. *EGFP* knockout efficiency was determined by FACS 6 days after transfection. Bars represent mean ± SEM from three independent experiments.

treatment, and nearly 50% of the control after 3 days. Longer treatment with ASV led to further reduction of the *EGFP* knockout cell fraction, which could be attributed to extended ASV treatment, progressive degradation of the input NC-SMASh Cas9 expression plasmid, or a combination of both. Taken together, these results demonstrate the rapid recovery of functional Cas9 following ASV removal, and this reversible control of the Cas9 activity is time-dependent.

Increased Gene Editing Specificity of NC-SMASh Cas9

The ability to modulate the intracellular Cas9 level allows us to address whether NC-SMASh Cas9 improves the specificity of gene editing. To restrict the production and accumulation of Cas9 protein, HEK293T cells were transfected with the expression plasmids for Cas9 and sgRNAs targeting different genomic loci in the presence of 20 μM ASV to suppress Cas9 accumulation, followed by ASV removal 24 h later to restore Cas9 for an additional 24 h. The validated on-target and off-target sites for each sgRNA were then PCR amplified and subjected to deep sequencing to quantify the indel-forming efficiency. The on-target loci we analyzed include the *WAS*, *VEGFA* (site 3), and *EMX1* genes.[39,40] We observed that WT Cas9 and NC-SMASh Cas9 showed a similar targeting specificity (on/off target ratio) in the absence of ASV (Figure S2). Removing ASV in cells expressing NC-SMASh Cas9 restored ∼50% of the gene editing efficiency at the on-target site relative to the control without ASV treatment (Figures 5A–5C). The efficiency of indel formation at the off-target sites was also reduced at the same time, but to a greater extent than the reduction at the on-target sites (Figures 5A–5C). Overall, the gene editing specificity with NC-SMASh Cas9 was enhanced relative to WT Cas9 at all three loci, by 1.5- to 3.1-fold for the *WAS* locus, 1.7- to 8.7-fold for the *VEGFA* locus, and 1.4- to 3.7-fold for the *EMX1* locus (Figure 5D). Thus, by adjusting the dose and duration of Cas9 exposure via the SMASh system, the specificity of gene editing is improved.

Because of the improved targeting fidelity of NC-SMASh Cas9, we examined whether this system could be applied to rationally designed

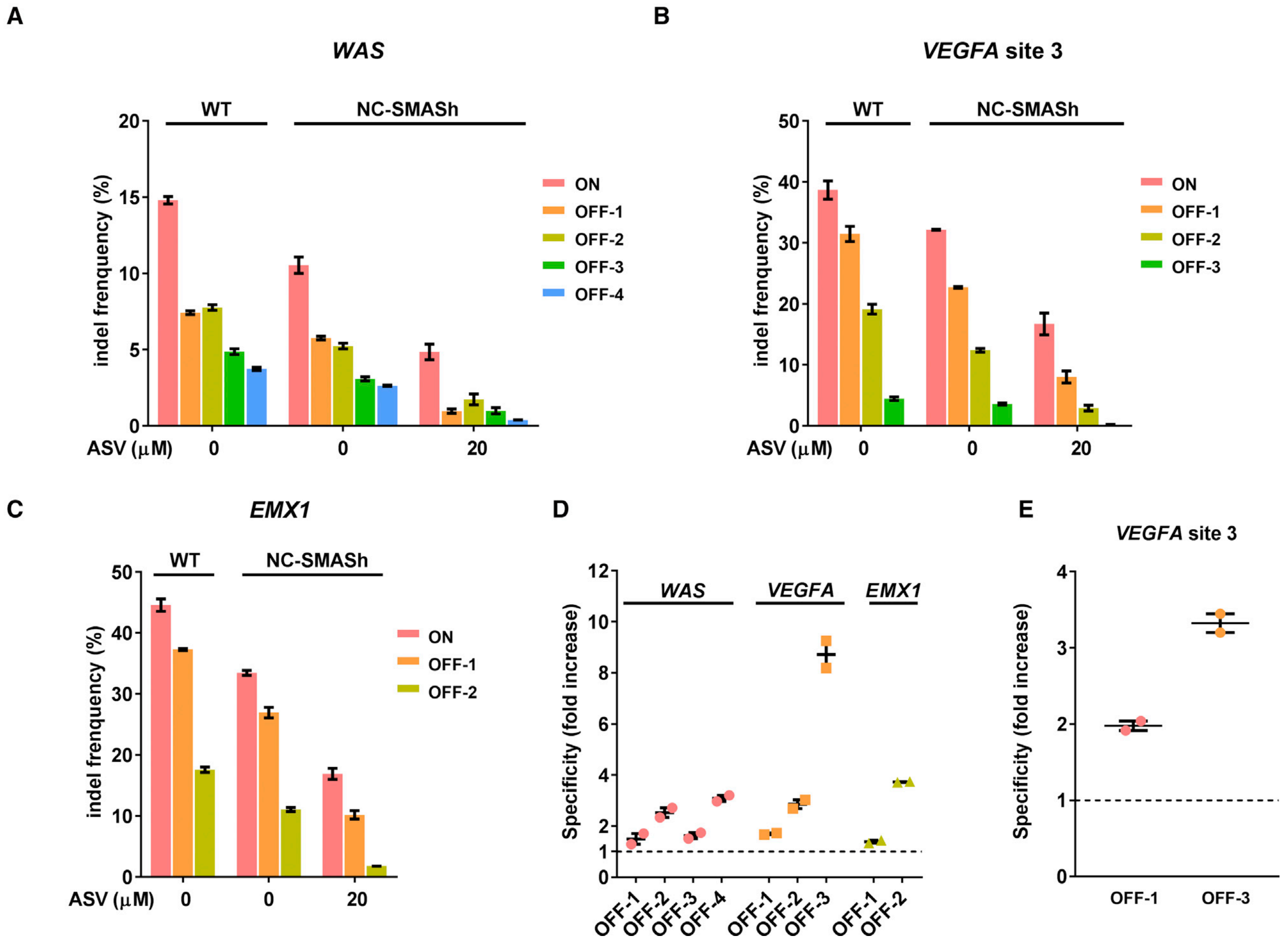

Figure 5. Improved Genome Editing Specificity by NC-SMASh Cas9

(A–C) Temporal control of NC-SMASh Cas9 for modulating gene editing specificity. Cas9 expression was initially suppressed by 20 μM ASV in HEK293T cells co-transfected with the expression plasmids for NC-SMASh Cas9 and the sgRNA targeting the genetic locus indicated, and it was restored 24 h later by ASV removal. Indel-forming efficiencies at the *WAS* (A), *VEGFA* (B), and *EMX1* (C) loci and their respective off-target sites were measured using deep sequencing. (D) Fold increase in specificity was determined based on the data in (A)–(C). Specificity was determined by dividing the indel frequency at each on-target site by those at the off-target sites. Fold increase in specificity of NC-SMASh Cas9 over WT Cas9 was then calculated for each off-target site. The data above dashed line indicates an increase in specificity (on/off > 1). Data are presented as mean ± SEM from two independent deep-sequencing experiments. (E) Increased targeting specificity with NC-SMASh eSpCas9. HEK293T cells were co-transfected with plasmids expressing the sgRNA for the *VEGFA* site 3 and either eSpCas9 or NC-SMASh eSpCas9 in the presence of 20 μM ASV as described above. On-target and off-target sites were PCR amplified and subjected to deep sequencing. Increase in specificity was determined as described in (D). Values are mean ± SEM from two independent experiments.

Cas9 variants such as eSpCas9 with enhanced specificity.[40] Attachment of the SMASh tag to both N and C termini of eSpCas9 (NC-SMASh eSpCas9) led to a similar gene editing efficiency as parental eSpCas9 at both the *VEGFA* and *EMX1* loci (Figure S3). This gene editing activity was abolished by the treatment with 20 μM ASV for 48 h. Similar to NC-SMASh Cas9, a significant portion of the gene editing activity of NC-SMASh eSpCas9 was restored when ASV was removed after 24 h of treatment (Figure S3). Although the parental eSpCas9 was shown to cleave some *EMX1* and *VEGFA* off-target sites at reduced efficiencies,[40] two *VEGFA* off-target sites, OFF-1 and OFF-3, were cleaved at similar efficiencies by both WT Cas9 and eSpCas9 (Figure S4). We examined the extent to which the two SMASh tags limited the off-target activity of eSpCas9 at these two *VEGFA* off-target sites. By suppressing the NC-SMASh eSpCas9 activity initially with ASV followed by ASV removal 24 h later in transfected HEK293T cells, the gene editing activity at both OFF-1 and OFF-3 was reduced, with a 2.0- and 3.3-fold improvement in the specificity, respectively (Figure 5E; Figure S4). Collectively, our data indicate that the genome editing specificity of both WT Cas9 and eSpCas9 can be improved by the ASV-controlled SMASh tag.

DISCUSSION

In this study, we fused Cas9 with the SMASh tag consisting of a protease domain and a degron domain from HCV. The presence of the

SMASh tag allows tight control of the Cas9 stability by ASV, a clinically approved HCV protease inhibitor. We showed that SMASh tag fusion did not significantly affect the gene editing activity of the modified Cas9 when compared with WT Cas9 in the absence of ASV. ASV administration led to selective degradation of newly synthesized Cas9 tagged with SMASh without affecting the stability of WT Cas9. Control of the Cas9 stability by ASV directly affected its gene editing efficiency. We also showed that suppression of the Cas9 protein level could be reversed by removing ASV, leading to a rapid restoration of the gene editing activity. Finally, we showed that by limiting the duration of Cas9 expression, the specificity of gene editing was improved. These results demonstrate the feasibility of using a clinically approved drug to control gene editing via the modulation of the Cas9 stability.

One commonly used approach for genome editing is through transfection of the expression plasmids for sgRNA and Cas9. Although effective, this approach usually leads to excessive production of the sgRNA and Cas9, but fails to promote the gene editing efficiency beyond certain threshold levels. This is consistent with our observation that the efficiency of indel formation was not always in proportion to the Cas9 level in transfected cells (Figures 1B, 2A, and 2B). A case in point is that C-SMASh Cas9-expressing cells treated with 500 nM ASV showed a dramatically reduced Cas9 level but continued to exhibit robust gene editing activity when compared with the control without ASV (Figures 2A and 2B). One drawback of excessive Cas9 production is the increased risk of off-target cleavage that could induce gene mutation or genome instability. Since cleavage of off-target sites by Cas9 presumably occurs less efficiently than of on-target sites due to lower binding affinity of CRISPR-Cas9 to off-target sites, a reduction in the Cas9 level could favor preferential binding of CRISPR-Cas9 to on-target sites. This is consistent with our data that genome editing specificity was elevated through the control of the Cas9 stability by ASV. Our study only tested limited ASV concentrations and duration of treatment. It is likely, however, that administration of ASV will need to be optimized for each individual case, depending on the target sequence, the binding affinity of the CRISPR-Cas9 complex to the target sequence, and the level of CRISPR-Cas9 in the cell.

To increase the targeting specificity of CRISPR-Cas9, Cas9 variant eSpCas9 was designed by introducing mutations into the REC3 domain based on structural studies.[40] Although eSpCas9 maintained efficient gene editing activity at several on-target sites tested, its activity was significantly reduced at some genomic loci, such as *WAS* and *TCF7L2* genes (Figure S5). This reduction could be due to an altered interaction between the guide RNA and eSpCas9 or specific chromatin associated with these genomic loci.[41] To perform gene editing efficiently in these loci, Cas9 rather than eSpCas9 may still be the preferred nuclease to use, and SMASh-tagged Cas9 will have the advantage of enhanced specificity. One major feature of eSpCas9 is its ability to reduce the off-target effect. However, not all off-target cleavages were eliminated by eSpCas9 as demonstrated by the two off-target sites for the *VEGF* sgRNA (Figure S4). We showed that by controlling the level of eSpCas9 with the SMASh tag, we enhanced the editing specificity of the *VEGF* sgRNA by reducing its cleavage at the two off-target sites (Figure 5E). Our study therefore reinforces the importance of modulating the stability of Cas9 and its variants to reduce the off-target effect.

Maji et al.[36] have reported recently the identification for the first time of a small molecule inhibitor, BRD0539, for Cas9. They showed that BRD0539 did not interfere with the formation of the CRISPR-Cas9 complex, but it inhibited the binding of the complex to its genomic target. In contrast, SMASh-tagged Cas9 described herein is regulated through the control of Cas9 degradation with a small molecule. Since these two systems modulate the Cas9 activity through different mechanisms, it would be interesting to determine whether potential synergies exist between them to better control the efficiency and specificity of gene editing. To date, CRISPR-Cas9 has become not just a genome editing tool, but it also has been developed into a versatile tool for base editing, gene activation or repression, epigenetic modulation, chromatin topology manipulation, and imaging.[5,42] We anticipate that the addition of the system described in our study could expand further the application of CRISPR-Cas9-based technologies across numerous dimensions in diverse organisms.

MATERIALS AND METHODS

Plasmid Construction

The human codon-optimized Cas9 plasmid phCas9[10] (Addgene, Cambridge, MA, USA) was used as WT Cas9. The U6 promoter and sgRNA scaffold were PCR amplified from PX330[8] and cloned into pBluescript SK(−) vector. A pair of annealed sgRNA oligonucleotides was cloned into the scaffold via BbsI sites. All sgRNA sequences are listed in Table S1.

To fuse a SMASh tag to the C terminus of Cas9, a 9,220-bp DNA fragment was isolated from phCas9 by AgeI and EcoRI digestion. The 939-bp self-cleaved NS3pro-NS4A fragment was PCR amplified from pCS6-YFP-SMASh (Addgene)[37] with FP1 and RP1 and digested with Nhe1 and Agel. The 341-bp split-Cas9 fragment with a C-terminal nuclear localization signal was PCR amplified from phCas9 with FP2 and RP2 and digested with EcoRI and NheI. These three fragments were then ligated together to generate pC-SMASh-Cas9.

To add the second SMASh tag to the N terminus of C-SMASh Cas9, a 275-bp N-terminal fragment of Cas9 was PCR amplified from pC-SMASh-Cas9 with FP3 and RP3 and digested with Xho1 and Sbf1. A 931-bp SMASh tag was PCR amplified from pCS6-SMASh-YFP (Addgene) with FP4 and RP4 and digested with Xba1 and Xho1. These two fragments were then ligated with the 10,184-bp fragment isolated from pC-SMASh-Cas9 digested with XbaI and SbfI to generate pNC-SMASh Cas9.

To construct pNC-SMASh-eSpCas9, a 1,120-bp DNA fragment of eSpCas9[40] containing all three mutations was synthesized as a gBlock and subcloned into pCR-BluntII-TOPO (Life Technologies, Carlsbad, CA, USA). A 1,082-bp fragment was then isolated from this plasmid

by PciI and XhoI digestion. A 6,888-bp fragment with XbaI and XhoI digestion of pNC-SMASh Cas9 and a 3,379-bp fragment with XbaI and PciI digestion of pNC-SMASh Cas9 were isolated. These three fragments were then ligated together. All primers used for plasmid construction are listed in Table S2.

Cell Culture and Transfection

HEK293T cells (CRL 3216, ATCC, Manassas, VA, USA) were maintained in high-glucose Dulbecco's modified Eagle's medium (DMEM) (Lonza, Allendale, NJ, USA) supplemented with 10% fetal bovine serum (HyClone, Logan, UT, USA) and 1% penicillin/streptomycin (Life Technologies). Cells were passaged when they reached ~80%–90% confluency. ASV (MedChemExpress, Monmouth Junction, NJ, USA) was dissolved in dimethyl sulfoxide (DMSO, JT Baker, Phillipsburg, NJ, USA) as a stock of 10 mM and diluted into culture medium to reach the desired final concentrations. DMSO without ASV was used as a negative control.

DNA transfection was performed with Lipofectamine 3000 (Life Technologies) when cells reached 40% confluence in 48-well plates according to the manufacturer's instruction. Briefly, 200 ng of Cas9 expression plasmid and 67 ng of sgRNA expression plasmid were co-transfected into the cells. To generate a stable cell line that constitutively expresses EGFP, HEK293T cells were plated at a density of 2×10^6/10-cm plate and co-transfected with 12 μg of pHIV7/PGK-GFP, 12 μg of pCgp, 4 μg of pRev-2, and 2 μg of pCMV-G by calcium phosphate co-precipitation 24 h after seeding.[43] Infectious virus was collected 48 h post-transfection and used to transduce HEK293T cells for 6 h in the presence of 4 μg/mL Polybrene. GFP-positive cells were sorted by FACS 48 h after transduction, serially diluted, and individual clones were isolated after a 2-week expansion.

Evaluation of Gene Editing Efficiency Using the Surveyor Assay

HEK293T cells were co-transfected with Cas9 and CRISPR expression plasmids as described above. After 48 h, the genomic DNA was isolated using the Epicenter QuickExtract solution (Epicenter Biotechnologies, Madison, WI, USA) and subjected to PCR amplification by Hotstar Taq (QIAGEN, Germantown, MD, USA) using primers flanking the CRISPR-Cas9 cleavage site (Table S3). The amplicons were denatured and reannealed, followed by digestion with the Surveyor nuclease (Integrated DNA Technologies, Skokie, IL, USA) for 1 h at 42°C. To calculate the efficiency of indel formation, digested products were separated on a 2% agarose gel, stained, and quantified with ImageJ software. The percentage of indel was calculated using the following equation: % indel formation = $100 \times [1 - (1 - \text{fraction cleaved})^{1/2}]$; the fraction cleaved = $100 \times$ sum of the cleavage product peak/cleavage product + parent peak.

EGFP Disruption Assay

HEK293T-EGFP cells generated by lentiviral transduction were transfected with a plasmid for a sgRNA targeting the *EGFP* gene along with the relevant Cas9 expression plasmid (WT Cas9, C-SMASh Cas9, or NC-SMASh Cas9) using Lipofectamine 3000. To compare the frequency of *EGFP* disruption under different conditions of ASV treatment, the fluorescence signal was either photographed under a Zeiss Axio Imager microscope (Carl Zeiss, San Diego, CA, USA) or analyzed using flow cytometry 6 days after transfection. For imaging, cells were re-plated on glass-bottom plates (Celltreat, Pepperell, MA, USA). Both differential interference contrast (DIC) images and EGFP fluorescence images were captured and analyzed using ZEN 2012 software. To quantify the loss of EGFP expression, transfected cells were trypsinized, resuspended in PBS containing 2% BSA, and analyzed on an Accuri C6 flow cytometer (BD Biosciences, Ann Arbor, MI, USA).

Western Blot Analysis

HEK293T cells expressing Cas9 transiently with or without ASV treatment were harvested and lysed in Pierce radioimmunoprecipitation assay (RIPA) buffer (Thermo Fisher Scientific, Waltham, MA, USA) supplemented with 1× HALT protease inhibitor cocktail (Thermo Fisher Scientific) and 5 mM EDTA. Protein concentrations were determined using a Pierce bicinchoninic acid (BCA) protein assay kit (Thermo Fisher Scientific). Approximately 20 μg of protein lysate was separated on a NuPAGE 4%–12% Bis-Tris gel (Invitrogen, Carlsbad, CA, USA) with 1× NuPAGE SDS running buffer, transferred to polyvinylidene fluoride (PVDF) membranes (Bio-Rad, Hercules, CA, USA), and blotted with a mouse anti-GAPDH antibody (1:2,000, GeneTex, Irvine, CA, USA) or a mouse anti-Cas9 antibody (1:1,000, Novus Biologicals, Littleton, CO, USA). After washing, the membranes were incubated in horseradish peroxidase-conjugated anti-mouse immunoglobulin G (IgG; 1:5,000, Thermo Fisher Scientific) at room temperature for 1 h, followed by exposure to enhanced chemiluminescence (ECL) substrate (Thermo Fisher Scientific). Membranes were visualized using a ChemiDoc imaging system (Bio-Rad).

Deep Sequencing of Potential Off-Target Sites

To detect indel induced by CRISPR-Cas9 editing, the genomic DNA was isolated and the locus of interest was PCR amplified using Q5 High-Fidelity DNA polymerase (New England Biolabs, Ipswich, MA, USA). The primers are listed in Table S4. Sample-specific barcodes were attached to the amplicons in this PCR step using forward primers with index sequences on the 5′ end. Following purification of the PCR products with NucleoSpin gel and a PCR Clean-up Kit (Macherey-Nagel, Duren, Germany), 5 ng of purified DNA from each PCR reaction was pooled. The sequencing libraries were prepared using a KAPA HyperPrep kit (Kapa Biosystems, catalog no. KR0961) according to the manufacturer's instructions. The libraries were validated with the Agilent Bioanalyzer DNA high-sensitivity DNA kit (Agilent, catalog no. 5067-4627) and quantified using Qubit and qPCR. The libraries were sequenced on an Illumina HiSeq 2500 with SBS (sequencing by synthesis) v4 reagent in the paired-end mode of 101 cycles of read 1, 7 cycles of the index read, and 101 cycles of read 2. The Real Time Analysis (RTA) 2.2.38 software was used to process the image analysis and base calling. Indels were calculated using CRISPResso software.[44]

SUPPLEMENTAL INFORMATION

Supplemental Information can be found online at https://doi.org/10.1016/j.omtn.2019.12.026.

AUTHOR CONTRIBUTIONS

Y.W., F.K., and J.-K.Y. conceived and designed experiments; L.Y. analyzed deep-sequencing data; Y.W. and T.C. performed the experiments; Y.W. provided data analysis and interpretation; Y.W. and J.-K.Y. wrote the manuscript.

CONFLICTS OF INTEREST

The authors declare no competing interests.

ACKNOWLEDGMENTS

Research reported in this publication included work performed in the Integrative Genomics Core and Analytical Cytometry Core supported by the National Cancer Institute of the National Institutes of Health under award no. P30CA033572. The content is solely the responsibility of the authors and does not necessarily represent the official views of the National Institutes of Health. This work was supported by a grant from the Wanek Innovation Program.

REFERENCES

1. Barrangou, R., Fremaux, C., Deveau, H., Richards, M., Boyaval, P., Moineau, S., Romero, D.A., and Horvath, P. (2007). CRISPR provides acquired resistance against viruses in prokaryotes. Science *315*, 1709–1712.
2. Gasiunas, G., Barrangou, R., Horvath, P., and Siksnys, V. (2012). Cas9-crRNA ribonucleoprotein complex mediates specific DNA cleavage for adaptive immunity in bacteria. Proc. Natl. Acad. Sci. USA *109*, E2579–E2586.
3. Horvath, P., and Barrangou, R. (2010). CRISPR/Cas, the immune system of bacteria and archaea. Science *327*, 167–170.
4. Knott, G.J., and Doudna, J.A. (2018). CRISPR-Cas guides the future of genetic engineering. Science *361*, 866–869.
5. Adli, M. (2018). The CRISPR tool kit for genome editing and beyond. Nat. Commun. *9*, 1911.
6. Doudna, J.A., and Charpentier, E. (2014). Genome editing. The new frontier of genome engineering with CRISPR-Cas9. Science *346*, 1258096.
7. Hsu, P.D., Lander, E.S., and Zhang, F. (2014). Development and applications of CRISPR-Cas9 for genome engineering. Cell *157*, 1262–1278.
8. Cong, L., Ran, F.A., Cox, D., Lin, S., Barretto, R., Habib, N., Hsu, P.D., Wu, X., Jiang, W., Marraffini, L.A., and Zhang, F. (2013). Multiplex genome engineering using CRISPR/Cas systems. Science *339*, 819–823.
9. Jinek, M., Chylinski, K., Fonfara, I., Hauer, M., Doudna, J.A., and Charpentier, E. (2012). A programmable dual-RNA-guided DNA endonuclease in adaptive bacterial immunity. Science *337*, 816–821.
10. Mali, P., Yang, L., Esvelt, K.M., Aach, J., Guell, M., DiCarlo, J.E., Norville, J.E., and Church, G.M. (2013). RNA-guided human genome engineering via Cas9. Science *339*, 823–826.
11. Hsu, P.D., Scott, D.A., Weinstein, J.A., Ran, F.A., Konermann, S., Agarwala, V., Li, Y., Fine, E.J., Wu, X., Shalem, O., et al. (2013). DNA targeting specificity of RNA-guided Cas9 nucleases. Nat. Biotechnol. *31*, 827–832.
12. Pattanayak, V., Lin, S., Guilinger, J.P., Ma, E., Doudna, J.A., and Liu, D.R. (2013). High-throughput profiling of off-target DNA cleavage reveals RNA-programmed Cas9 nuclease specificity. Nat. Biotechnol. *31*, 839–843.
13. Fu, Y., Foden, J.A., Khayter, C., Maeder, M.L., Reyon, D., Joung, J.K., and Sander, J.D. (2013). High-frequency off-target mutagenesis induced by CRISPR-Cas nucleases in human cells. Nat. Biotechnol. *31*, 822–826.
14. Yen, S.T., Zhang, M., Deng, J.M., Usman, S.J., Smith, C.N., Parker-Thornburg, J., Swinton, P.G., Martin, J.F., and Behringer, R.R. (2014). Somatic mosaicism and allele complexity induced by CRISPR/Cas9 RNA injections in mouse zygotes. Dev. Biol. *393*, 3–9.
15. Wang, H., Yang, H., Shivalila, C.S., Dawlaty, M.M., Cheng, A.W., Zhang, F., and Jaenisch, R. (2013). One-step generation of mice carrying mutations in multiple genes by CRISPR/Cas-mediated genome engineering. Cell *153*, 910–918.
16. Tu, Z., Yang, W., Yan, S., Yin, A., Gao, J., Liu, X., Zheng, Y., Zheng, J., Li, Z., Yang, S., et al. (2017). Promoting Cas9 degradation reduces mosaic mutations in non-human primate embryos. Sci. Rep. *7*, 42081.
17. Qi, L.S., Larson, M.H., Gilbert, L.A., Doudna, J.A., Weissman, J.S., Arkin, A.P., and Lim, W.A. (2013). Repurposing CRISPR as an RNA-guided platform for sequence-specific control of gene expression. Cell *152*, 1173–1183.
18. Peters, J.M., Colavin, A., Shi, H., Czarny, T.L., Larson, M.H., Wong, S., Hawkins, J.S., Lu, C.H.S., Koo, B.M., Marta, E., et al. (2016). A comprehensive, CRISPR-based functional analysis of essential genes in bacteria. Cell *165*, 1493–1506.
19. Rock, J.M., Hopkins, F.F., Chavez, A., Diallo, M., Chase, M.R., Gerrick, E.R., Pritchard, J.R., Church, G.M., Rubin, E.J., Sassetti, C.M., et al. (2017). Programmable transcriptional repression in mycobacteria using an orthogonal CRISPR interference platform. Nat. Microbiol. *2*, 16274.
20. Gilbert, L.A., Larson, M.H., Morsut, L., Liu, Z., Brar, G.A., Torres, S.E., Stern-Ginossar, N., Brandman, O., Whitehead, E.H., Doudna, J.A., et al. (2013). CRISPR-mediated modular RNA-guided regulation of transcription in eukaryotes. Cell *154*, 442–451.
21. Chavez, A., Scheiman, J., Vora, S., Pruitt, B.W., Tuttle, M., P R Iyer, E., Lin, S., Kiani, S., Guzman, C.D., Wiegand, D.J., et al. (2015). Highly efficient Cas9-mediated transcriptional programming. Nat. Methods *12*, 326–328.
22. Rodríguez-Rodríguez, D.R., Ramírez-Solís, R., Garza-Elizondo, M.A., Garza-Rodríguez, M.L., and Barrera-Saldaña, H.A. (2019). Genome editing: a perspective on the application of CRISPR/Cas9 to study human diseases (Review). Int. J. Mol. Med. *43*, 1559–1574.
23. Chew, W.L. (2018). Immunity to CRISPR Cas9 and Cas12a therapeutics. Wiley Interdiscip. Rev. Syst. Biol. Med. *10*, e1408.
24. Wagner, D.L., Amini, L., Wendering, D.J., Burkhardt, L.M., Akyüz, L., Reinke, P., Volk, H.D., and Schmueck-Henneresse, M. (2019). High prevalence of *Streptococcus pyogenes* Cas9-reactive T cells within the adult human population. Nat. Med. *25*, 242–248.
25. Foss, D.V., Hochstrasser, M.L., and Wilson, R.C. (2019). Clinical applications of CRISPR-based genome editing and diagnostics. Transfusion *59*, 1389–1399.
26. Cao, J., Wu, L., Zhang, S.M., Lu, M., Cheung, W.K., Cai, W., Gale, M., Xu, Q., and Yan, Q. (2016). An easy and efficient inducible CRISPR/Cas9 platform with improved specificity for multiple gene targeting. Nucleic Acids Res. *44*, e149.
27. Davis, K.M., Pattanayak, V., Thompson, D.B., Zuris, J.A., and Liu, D.R. (2015). Small molecule-triggered Cas9 protein with improved genome-editing specificity. Nat. Chem. Biol. *11*, 316–318.
28. Zetsche, B., Volz, S.E., and Zhang, F. (2015). A split-Cas9 architecture for inducible genome editing and transcription modulation. Nat. Biotechnol. *33*, 139–142.
29. Liu, K.I., Ramli, M.N., Woo, C.W., Wang, Y., Zhao, T., Zhang, X., Yim, G.R., Chong, B.Y., Gowher, A., Chua, M.Z., et al. (2016). A chemical-inducible CRISPR-Cas9 system for rapid control of genome editing. Nat. Chem. Biol. *12*, 980–987.
30. Maji, B., Moore, C.L., Zetsche, B., Volz, S.E., Zhang, F., Shoulders, M.D., and Choudhary, A. (2017). Multidimensional chemical control of CRISPR-Cas9. Nat. Chem. Biol. *13*, 9–11.
31. Nihongaki, Y., Kawano, F., Nakajima, T., and Sato, M. (2015). Photoactivatable CRISPR-Cas9 for optogenetic genome editing. Nat. Biotechnol. *33*, 755–760.
32. Nihongaki, Y., Otabe, T., and Sato, M. (2018). Emerging approaches for spatiotemporal control of targeted genome with inducible CRISPR-Cas9. Anal. Chem. *90*, 429–439.
33. Zhou, X.X., Zou, X., Chung, H.K., Gao, Y., Liu, Y., Qi, L.S., and Lin, M.Z. (2018). A single-chain photoswitchable CRISPR-Cas9 architecture for light-inducible gene editing and transcription. ACS Chem. Biol. *13*, 443–448.
34. Pawluk, A., Amrani, N., Zhang, Y., Garcia, B., Hidalgo-Reyes, Y., Lee, J., Edraki, A., Shah, M., Sontheimer, E.J., Maxwell, K.L., et al. (2016). Naturally occurring off-switches for CRISPR-Cas9. Cell *167*, 1829–1838.e9.

35. Shin, J., Jiang, F., Liu, J.J., Bray, N.L., Rauch, B.J., Baik, S.H., Nogales, E., Bondy-Denomy, J., Corn, J.E., and Doudna, J.A. (2017). Disabling Cas9 by an anti-CRISPR DNA mimic. Sci. Adv. *3*, e1701620.

36. Maji, B., Gangopadhyay, S.A., Lee, M., Shi, M., Wu, P., Heler, R., Mok, B., Lim, D., Siriwardena, S.U., Paul, B., et al. (2019). A high-throughput platform to identify small-molecule inhibitors of CRISPR-Cas9. Cell *177*, 1067–1079.e19.

37. Chung, H.K., Jacobs, C.L., Huo, Y., Yang, J., Krumm, S.A., Plemper, R.K., Tsien, R.Y., and Lin, M.Z. (2015). Tunable and reversible drug control of protein production via a self-excising degron. Nat. Chem. Biol. *11*, 713–720.

38. Kim, S., Kim, D., Cho, S.W., Kim, J., and Kim, J.S. (2014). Highly efficient RNA-guided genome editing in human cells via delivery of purified Cas9 ribonucleoproteins. Genome Res. *24*, 1012–1019.

39. Wang, X., Wang, Y., Wu, X., Wang, J., Wang, Y., Qiu, Z., Chang, T., Huang, H., Lin, R.J., and Yee, J.K. (2015). Unbiased detection of off-target cleavage by CRISPR-Cas9 and TALENs using integrase-defective lentiviral vectors. Nat. Biotechnol. *33*, 175–178.

40. Slaymaker, I.M., Gao, L., Zetsche, B., Scott, D.A., Yan, W.X., and Zhang, F. (2016). Rationally engineered Cas9 nucleases with improved specificity. Science *351*, 84–88.

41. Jensen, K.T., Fløe, L., Petersen, T.S., Huang, J., Xu, F., Bolund, L., Luo, Y., and Lin, L. (2017). Chromatin accessibility and guide sequence secondary structure affect CRISPR-Cas9 gene editing efficiency. FEBS Lett. *591*, 1892–1901.

42. Tadic, V., Josipovic, G., Zoldos, V., and Vojta, A. (2019). CRISPR/Cas9-based epigenome editing: an overview of dCas9-based tools with special emphasis on off-target activity. Methods *164–165*, 109–119.

43. Lo, H.L., Chang, T., Yam, P., Marcovecchio, P.M., Li, S., Zaia, J.A., and Yee, J.K. (2007). Inhibition of HIV-1 replication with designed miRNAs expressed from RNA polymerase II promoters. Gene Ther. *14*, 1503–1512.

44. Pinello, L., Canver, M.C., Hoban, M.D., Orkin, S.H., Kohn, D.B., Bauer, D.E., and Yuan, G.C. (2016). Analyzing CRISPR genome-editing experiments with CRISPResso. Nat. Biotechnol. *34*, 695–697.

OMTN, Volume *19*

Supplemental Information

A Small Molecule-Controlled

Cas9 Repressible System

Youjun Wu, Lu Yang, Tammy Chang, Fouad Kandeel, and Jiing-Kuan Yee

Supplemental information

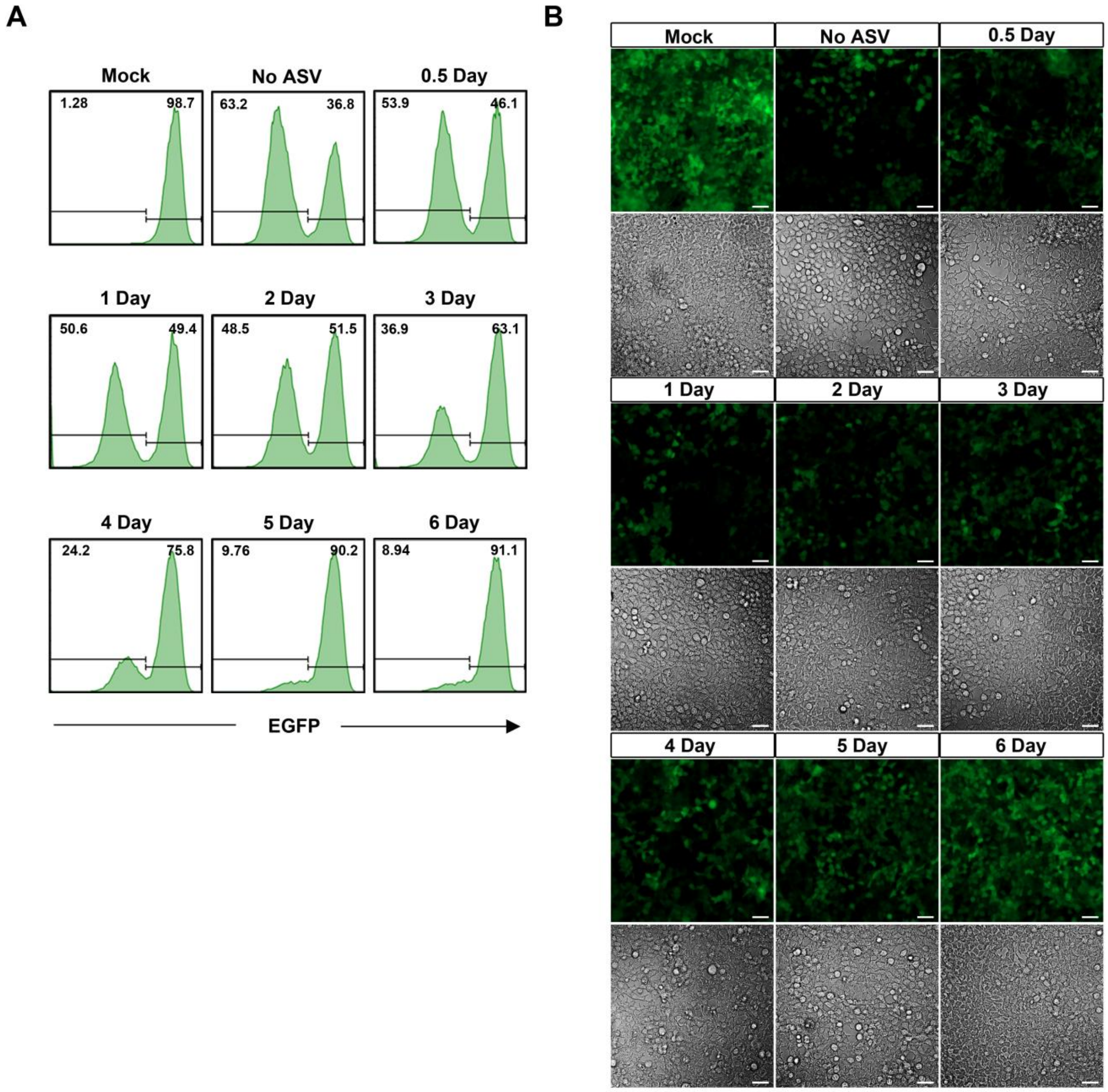

Figure S1. Restoration of the *EGFP* gene knockout activity following ASV removal. HEK293T cells co-transfected with plasmids expressing NC-SMASh Cas9 and a sgRNA against the *EGFP* gene in the presence of 20 µM ASV were washed at indicated time points. The *EGFP* gene knockout efficiency was measured by FACS in (A) and fluorescence microscopy in (B) 6 days post-transfection. Data shown is a representative of three independent experiments. Scale bar = 50 µm.

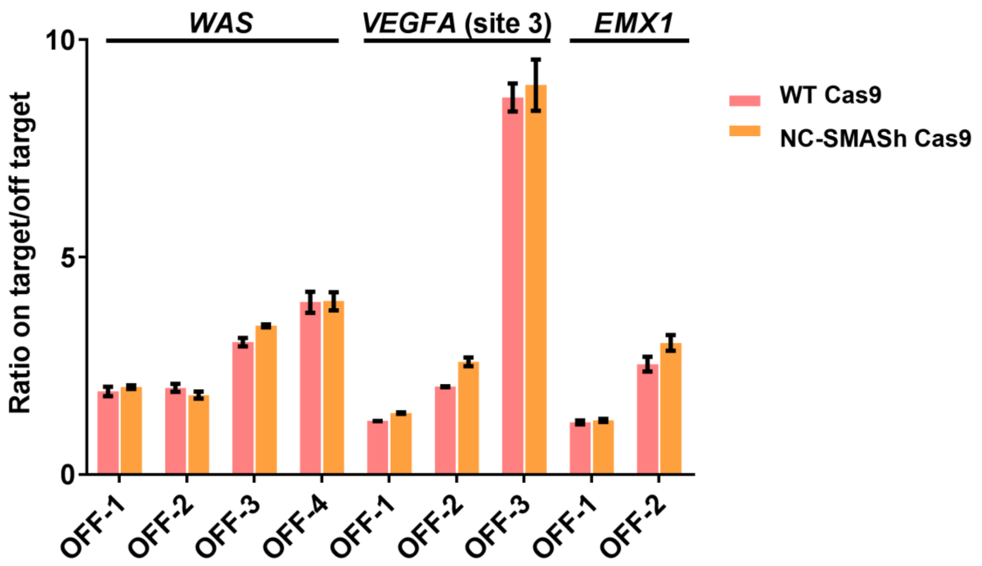

Figure S2. Similar targeting specificity between WT Cas9 and NC-SMASh Cas9 in the absence of ASV. HEK293T cells were co-transfected with plasmids expressing either WT Cas9 or NC-SMASh Cas9 and the sgRNA targeting the *WAS*, *VEGFA* or *EMX1* locus as indicated. On- and off-target sites were PCR amplified and subjected to deep sequencing. The on/off target site ratio was calculated with the indel frequency of each locus. Data represents mean ± SEM from two independent deep-sequencing experiments.

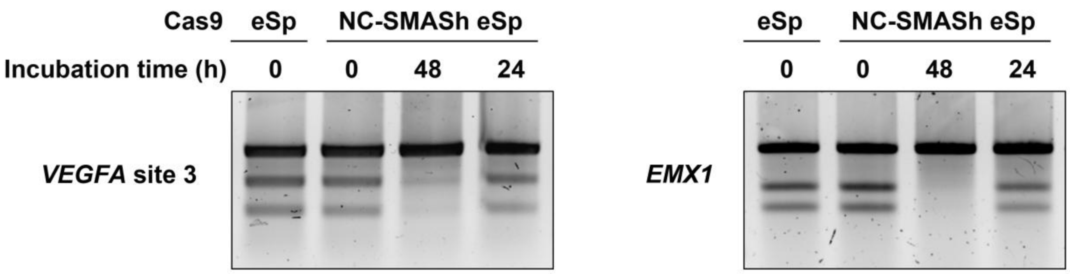

Figure S3. Reversible gene editing with NC-SMASh eSpCas9 in response to ASV treatment. HEK293T cells were co-transfected with plasmids expressing either eSpCas9 or NC-SMASh eSpCas9 and the sgRNA targeting the *VEGFA* site 3 or *EMX1* locus as indicated. The transfected cultures were treated with 20 µM ASV for 48 h or treated for the first 24 h followed by ASV removal and incubation for an additional 24 h in fresh medium without ASV. All cultures were harvested 48 h after transfection and the isolated genomic DNA was subjected to the Surveyor assay to measure indel formation.

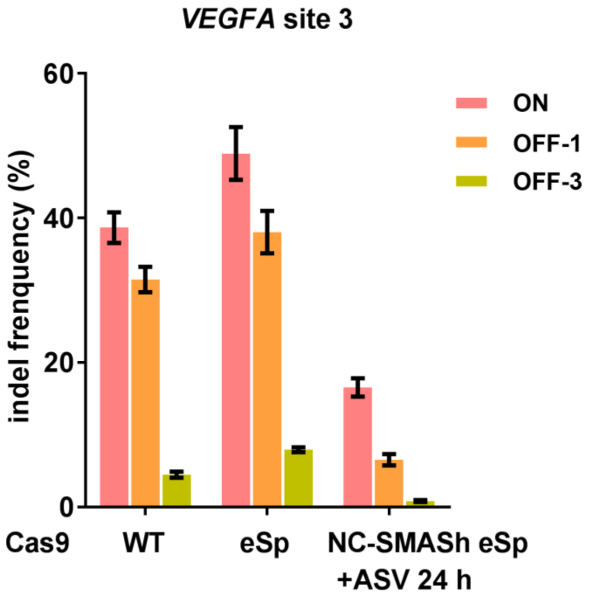

Figure S4. Increased targeting specificity of NC-SMASh Cas9. HEK293T cells were co-transfected with plasmids expressing WT Cas9, eSpCas9, or NC-SMASh eSpCas9 and the sgRNA targeting the *VEGFA* site 3 locus. Cells expressing NC-SMASh eSpCas9 were treated with 20 µM ASV immediately after transfection for 24 h followed by its removal and incubation in fresh medium for an additional 24 h. On- and off-target sites were PCR amplified and subjected to deep sequencing. Data represent mean ± SEM from two independent deep-sequencing experiments.

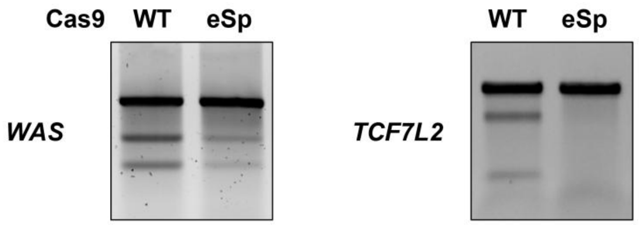

Figure S5. Genomic loci resistant to the cleavage by eSpCas9. HEK293T cells were co-transfected with plasmids expressing WT Cas9 or eSPCas9 and the sgRNA targeting either the *WAS* or *TCF7L2* locus. Surveyor assay was performed to detect indel formation.

Table S1. sgRNA sequences used in this paper.

Gene	Sequence	PAM
WAS	TGGATGGAGGAATGAGGAGT	TGG
PCSK1	ATAAGTGTGTGGTCCCAAGT	TGG
EGFP	GGCGAGGGCGATGCCACCTA	CGG
VEGFA	GGTGAGTGAGTGTGTGCGTG	TGG
EMX1	GAGTCCGAGCAGAAGAAGAA	GGG
TCF7L2	TATAATTTAATTGCCGTGTG	AGG

Table S2. Primers for plasmid construction

Plasmid	Primer set	Product size
C-SMASh Cas9	FP1: CAGCTAGCTAGCGATGAGATGGAAGAGTGCTC<br>RP1: ACAGCGACCGGTTCAGTAGAGAACCTCCCTGTC	939
	FP2: TCGAGCAAATAAGCGAATTCTCC<br>RP2: ACAGCGGCTAGCCACCTTCCTCTTCTTCTTGGGGTC	341
NC-SMASh Cas9	FP3: CAGAAGGCTCGAGGACAAGAAGTACTCCATTGG<br>RP3: CATTACTAAAGATCTCCTGCAGGTAGC	275
	FP4: TGCTCTAGAATGGATTACAAGGATGACGACG<br>RP4: CAGAAGGCTCGAGGCCCATTGAGCATGGCACG	931

Table S3. Primers used for Surveyor assay

Target Gene	Primer set	Expected PCR product (bp)	Expected cleaved products (bp)
WAS	FP: AAAGGAAGTTGGGCAGAGGTGAGT<br>RP:<br>CCATCGATTGTGTGTTGGATGGTCATGGAGGT	838	499; 339
PCSK1	FP: GCCCACACAAGAGAACCCTAA<br>RP: TTGAGCTCATCCCCTTCACAT	540	391; 149
VEGFA	FP: CTCACTTTGATGTCTGCAGGCCA<br>RP: CGAGGAGGGAGCAGGAAAGTGA	528	333; 195
EMX1	FP: CCATCCCCTTCTGTGAATGT<br>RP: GGAGATTGGAGACACGGAGA	639	272; 367
TCF7L2	FP: TCTTGCTTAGTCACTTTCTGTTTGAACA<br>RP: CAGTGAAGTGCCCAAGCTTCTCA	500	360; 140

Table S4. Primers used for deep-sequencing

Name	Gene	Target sequence	Primer set
WAS ON	*WAS*	TGGATGGAGGAATGAGGAGTTGG	FP:TGCGTGCTGATTCTTCCCTG<br>RP:TGCTCGTCCATCCACATACC
WAS OFF1	*MATN1-AS1*	TGGATGGAGGGATGAGGAGTGGG	FP:TGGCACATAGAGGAGCCTGA<br>RP:GGGGGCTGTTTGTTATGCAC
WAS OFF2	*OSBPL1A*	AGGAGGGAGGAATGGGGAGTTGG	FP:AGTAGGGTTAGGCCACGACA<br>RP:TTCCTATGCAACCATCACCA
WAS OFF3	*MYCN*	GAGGATGGGGAATGAGGAGTAGG	FP:TTGGGCAGGTGGGAGTCAAT<br>RP:CCACTCCTGTTGACCTGCTC
WAS OFF4	*CD70*	CGGACGGAGGAATGGGGAGTGGG	FP:GGCACTGGTCACCTGTTTGA<br>RP:TGAGAGGAGTGCTTGATGAAAAGA
VEGFA ON	*VEGFA* (site 3)	GGTGAGTGAGTGTGTGCGTGTGG	FP:GCGAGCAGCGTCTTCGAGAGTGAGGA<br>RP:GGAGAGGGACACACAGATCTATTGGAA
VEGFA OFF1	*MAX*	AGTGAGTGAGTGTGTGTGTGGGG	FP: AGGGGAGGGGGAAGTCACCGA<br>RP: GGAGGTGGTTCTTGCCCGTG
VEGFA OFF2	*CYTH4*	GCTGAGTGAGTGTATGCGTGTGG	FP: GCCCATTTCTCCTTTGAGGTTCATCC<br>RP: TGGTGGGGACAGCATGTGCAAG
VEGFA OFF3	*TMEM121 (abParts)*	GGTGAGTGAGTGTGTGTGTGAGG	FP: AGGGACCCCTCTGACAGACT<br>RP: AGCCCTCAGACTTCACATTC
EMX1 ON	*EMX1*	GAGTCCGAGCAGAAGAAGAAGGG	FP: CAAAGTACAAACGGCAGAAGC<br>RP: GTTGCCCACCCTAGTCATTG
EMX1 OFF1	*HCN1*	GAGTTAGAGCAGAAGAAGAAAGG	FP: TATCACCTATTTTTTCTGAGGGCTGCT<br>RP: GGGTTACAGAAAGAATAGGGGCTTATG
EMX1 OFF2	*MFAP1*	GAGTCTAAGCAGAAGAAGAAGAG	FP: CACGGCCTTTGCAAATAGAG<br>RP: GGCTTTCACAAGGATGCAGT

Mismathes are colored in red.
